# An integrated pre-clerkship curriculum to build cognitive medical schema: It’s not just about the content

**DOI:** 10.3389/fphys.2023.1148916

**Published:** 2023-03-16

**Authors:** Renée J. LeClair, Jennifer L. Cleveland, Kristin Eden, Andrew P. Binks

**Affiliations:** Department of Basic Science Education, Virginia Tech Carilion School of Medicine, Roanoke, VA, United States

**Keywords:** illness script, clinical reasoning, curricular design, specifications grading, concept map

## Abstract

Both physiology and pathophysiology are essential disciplines in health professional education however, clinicians do not use this knowledge in isolation. Instead, physicians use inter-disciplinary concepts embedded within integrated cognitive schema (illness scripts) established through experience/knowledge that manifest as expert-level thinking. Our goal was to develop a pre-clerkship curriculum devoid of disciplinary boundaries (akin to the physician’s illness script) and enhance learners’ clerkship and early clinical performance. As well as developing curricular content, the model considered non-content design elements such as learner characteristics and values, faculty and resources and the impact of curricular and pedagogical changes. The goals of the trans-disciplinary integration were to develop deep learning behaviors through, 1) developing of integrated, cognitive schema to support the transition to expert-level thinking, 2) authentic, contextualization to promote knowledge transfer to the clinical realm 3) allowing autonomous, independent learning, and 4) harnessing the benefits of social learning. The final curricular model was a case-based approach with independent learning of basic concepts, differential diagnosis and illness scripting writing, and concept mapping. Small-group classroom sessions were team-taught with basic scientists and physicians facilitating learners’ self-reflection and development of clinical reasoning. Specifications grading was used to assess the products (written illness scripts and concept maps) as well as process (group dynamics) while allowing a greater degree of learner autonomy. Although the model we adopted could be transferred to other program settings, we suggest it is critical to consider both content and non-content elements that are specific to the environment and learner.

## 1 Introduction

Both physiology and pathophysiology are essential disciplines in health professional education however, clinicians do not use this knowledge in isolation. For example, when a pulmonologist meets a patient with allergies, wheeze and labored breathing, her knowledge of physiology, immunology, pharmacology and epidemiology lead her to place asthma high on the differential diagnosis and inhaled steroids as a potential therapy. She does not however, compartmentalize her diagnostic process within these distinct disciplines but instead, she uses pertinent concepts from each that are embedded in an integrated cognitive schema of how asthma presents. Similarly, any patient characteristics that did not fit her cognitive schema of asthma (e.g., if the patient also had a low-grade fever) would catch her attention and cause her to consider an alternative diagnoses/management. This level of conceptual integration manifests as expert-level thinking.

The integration of seemingly independent concepts is embedded in *script theory* that states the expert’s organization of pre-compiled knowledge and experience helps them develop a cognitive script (or schema) of the environment around them (Shank and Abelson, 1977). This theory has been applied to both clinical reasoning and medical education as “illness scripts” (Charlin et al., 2007; Custers, 2015) that represent an experienced physician’s integration and organization of medical knowledge that gives rise to superior problem-solving, enhanced memory retrieval and expert-level reasoning. The pulmonologist’s cognitive schema of asthma became established with her experience with numerous asthmatic patients that allowed her to make robust conceptual connections outside of disciplinary silos. Meanwhile, without an organized illness script, the novice medical student will likely fail to fully comprehend the connections between symptomology and pertinent concepts; a failure that may have been inadvertently promoted by discipline-based education. To overcome this, our goal was to couple the use of well-established educational tools (illness scripts and concept maps) to interweave relevant basic and clinical sciences content to promote an early transition from novice-to expert-thinking (Djulbegovic et al., 2012; Kirch et al., 2013) and better prepare our students for clerkships and clinical reasoning.

To achieve our goal, we aimed to have learners establish learning behaviors that incorporated their existing and newly attained knowledge into learner generated illness scripts prologues (Lubarsky et al., 2015). To promote cognitive script formation, we integrated physiology with other medically-relevant content at a trans-disciplinary level (Harden, 2000) in a curriculum free of any disciplinary distinctions; akin to the expert’s illness scripts. This trans-disciplinary curricular approach required a structured framework to integrate multiple basic science disciplines and the clinical sciences (Edmondson, 1995) in authentic clinical scenarios. While practicing integration of their own cognitive scripts we also wanted our learners to simultaneously observe a physician’s selection, filtration and interpretation of clinical data to demonstrate the value of an expert’s integrated clinical reasoning process.

**FIGURE 1:**
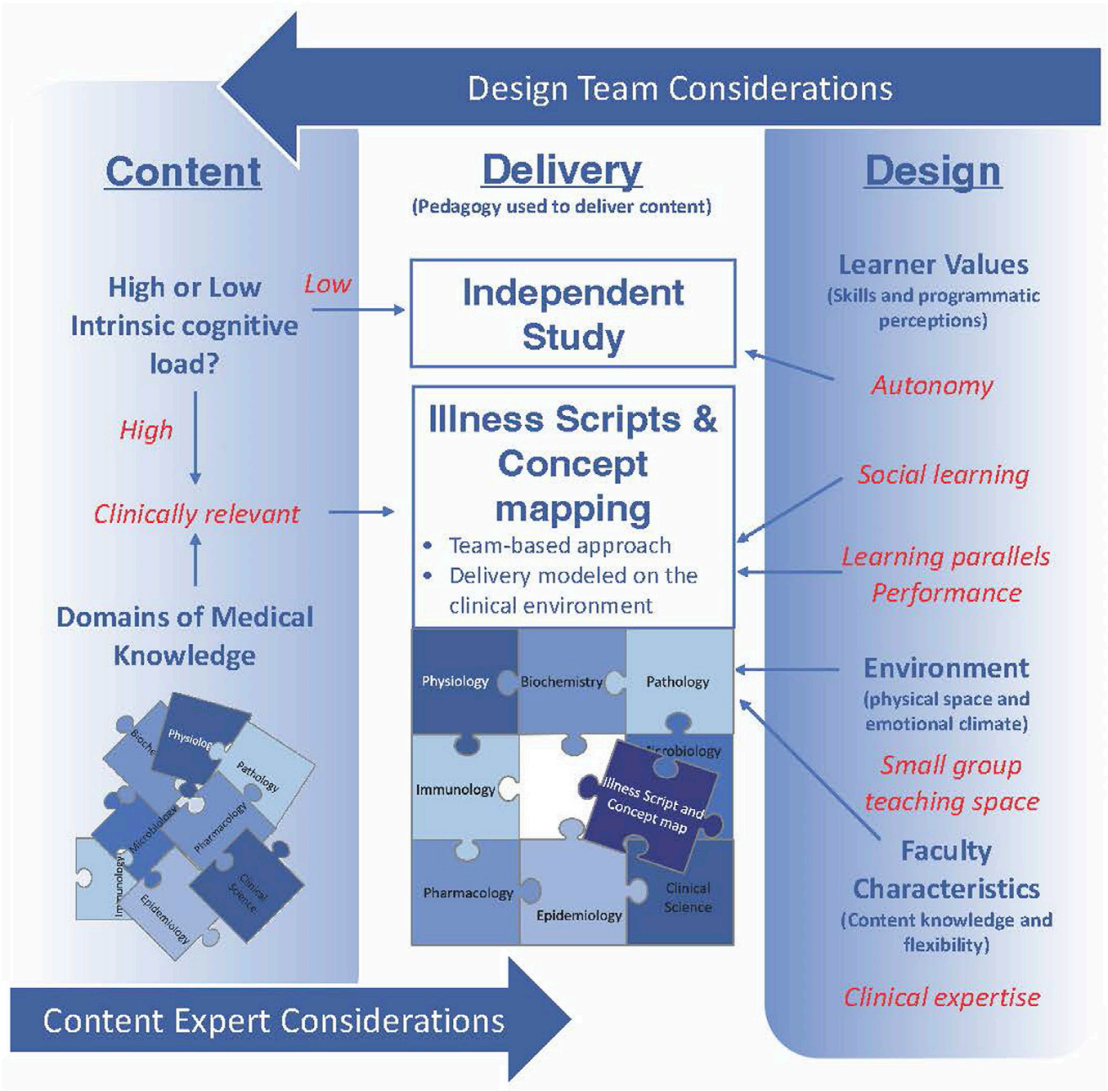
A schematic representation of elements to consider in the generation of an integrated curriculum. Beyond considering the specific content to include (left column) there are design elements that are integral to implementing a curriculum aligned with learner characteristics, resources and faculty (right column). Considering both content and design elements promotes selection of pedagogies (center column) that are best suited to achieve educational goals. Red text indicates elements specific to our model that may not be the same in other learning settings.

The benefits of using illness scripts or concepts maps in medical education as a way to integrate content has many reported benefits (Harden et al., 1984; Schmidt et al., 1996), however, these reports often describe difficulties toward implementation and numerous barriers (and solutions) have been described (E. National Academies of Sciences et al., 2018). In designing our model, we determined we not only needed to identify content (Figure 1, left column), but also select learning methods that would allow practice and visualization of integrated cognitive scripts. Perhaps more importantly, we considered curricular design aspects unrelated to content (Khalil and Elkhider, 2016) (Figure 1, right column). Including these design aspects would be critical to aligning a new learning process with the needs and characteristics of the learner.

## 2 Non-content considerations: The learner, the methods and the educator

By asking ourselves the following questions, we leveraged characteristics of our learners, addressed the extraneous cognitive load of novel pedagogies, considered the physical learning environment, and thoughtfully selected focused preparation materials and classroom educators.

### 2.1 Who are your learners?

Our target audience of 49 second-year medical students are veteran independent learners with access to numerous, board-exam orientated, extra-curricular resources and a preference for autonomous control of their basic science learning (Chen et al., 2019; Binks et al., 2021). Although this may contribute to declining class attendance in medical schools (AAMC, 2017) and an arising independent “parallel curriculum” (Coda, 2019), we saw the students’ ability to master content independently (at least sufficiently to pass board exams) as the opportunity to repurpose the classroom and reach a higher-level educational outcome. However, requiring learners to modify their independent and successful learning behaviors to any extent would require careful navigation and administrative support.

### 2.2 What are learners’ perceptions of teaching and learning?

Medical students’ knowledge of learning theory and active classroom instruction is generally low (Michaelsen and Sweet, 2008). With significant track-records of success using their established learning behaviors in traditional lecture-based delivery programs, change is usually perceived as unnecessary and met with resistance (Yengo-Kahn et al., 2017). Our model of integration represented a significant curricular change, so it was important to reassure the learners of the evidence-base and long-term benefits of the changes we were making. While the vast majority of the class understood and accepted our reasons for the change, some stakeholders remained vocally upset regardless of high levels of communication. In reflection, having a small number of learners express dissatisfaction is not uncommon nor unique to our permutation of curricular change (Bernier et al., 2000). However, interpreting student feedback related to active learning and avoiding over reacting to initial responses should guide, rather than reverse curricular progress (LeClair et al., 2018). By remaining attentive and demonstrably responsive to learner feedback and making changes where appropriate, we provided some reassurance. The opportunity to practice new learning methods with faculty feedback also helped gain trust and was an essential element to generating a positive learning environment.

### 2.3 Is the learning environment suited for the pedagogy?

We intentionally harnessed modern adult learners’ aptitude for social learning (Bandura and McClelland, 1977; Boysen et al., 2016; Binks et al., 2021). Although appearing juxtaposed to the preference for learning autonomy, adopting a small-group environment allowed learners to actively compare and integrate each individual’s baseline knowledge and collectively benefit from “the more knowledgeable other” (Vygotsky and Cole, 1978). We therefore planned activities for small groups (four learners) (Ambrose et al., 2010; Millis, 2014) who were sat at tables with a single computer and large monitor to serve as a focal point of the activity. To be effective however, a small group must adopt positive group dynamics in order to generate a supportive learning environment (Kitchen, 2012). Practicing developing team dynamic skills could therefore not only better prepare our learners for team-based clinical practice, but also enhance learning. Including assessment of group dynamics [assessed with an adapted rubric from Hopper et al (2020)]as well as content integration was therefore considered an important element to emphasize its immediate and long-term importance. We also needed our learners to integrate concepts in the manner in which they were going to use them professionally (Ambrose et al., 2010), i.e., the cognitive script we were asking them to generate would be applicable in their clerkships and clinical career. We therefore wanted to mimic the clinical environment as closely as possible and so adopted a case-based approach.

### 2.4 What do your learners value?

Faculty motivations for integrated activities must be articulated and aligned with the values of their learners (E. National Academies of Sciences et al., 2018). Our learners’ values of autonomous and social learning were incorporated by adopting a modified version of specifications grading [reviewed in (Nilson, 2015)]. This grading model has been shown to enhance behavior change, place value on the learning process and increase rigor (Nilson, 2015; Katzman et al., 2021). It also provides learner autonomy by allowing some level of non-mastery and opportunity to address deficits, thereby allowing the learner some degree of choice (i.e., autonomy) in their level of engagement. Specifications grading allowed us to incorporate assessment of group dynamics and process, thereby emphasizing our shared value of social learning and other core medical competencies (e.g., interpersonal communication and professionalism). Specifications grading however, relies on timely and comprehensive faculty feedback (Eppich et al., 2015) if students are to be able to address deficits in learning or group dynamics.

### 2.5 Do you have the design team and administrative support needed for a curricular shift?

Learners are not the only population to consider when making educational changes and faculty are usually the major barrier to curricular reform (Anderson, 1970; Bernier et al., 2000). Selection of the faculty educators needs to be carefully considered because of the impact they have on the learning environment. Regardless of organizational or departmental structure, the initial team should be small and be primarily comprised of existing faculty members who are savvy with curricular design, rather than just content expertise (Malik and Malik, 2011; Willey et al., 2018). In doing so, the curricular structure and classroom plan can be defined before recruitment of additional content experts to ensure consistent implementation and attainment of programmatic goals. Prior to delivery, we sent a voice-over-video to content experts explaining the rationale for change, the curriculum design and defining the classroom roles. Combined with strong administrative backing this helped address common concerns, clarify the expectations of faculty and the boundaries of their content. For busy clinicians or researchers, having a dedicated team focused on organization, presentation and continuity was a welcome change as it significantly reduced the time they needed to prepare.

### 2.6 What were the curricular elements needed to reach your goal?

Considering our educational goal and these instructional design elements, we determined our model needed to 1) promote development of integrated, cognitive schema to support the transition to expert-level thinking, 2) be authentic and contextual to promote engagement and knowledge transfer to the clinical realm 3) allow independent learning for continued board-exam success, and 4) harness the benefits of social learning.

## 3 Selecting classroom methods

The final curricular model, implemented in the second year of pre-clerkship education (Figure 1, center column), focused on an integrative, case-based approach with three elements, 1) independent learning of basic concepts, 2) differential diagnosis and illness scripting, and 3) concept mapping. To provide time for autonomous, independent learning, we reduced classroom hours from approximately 60 h to 16 h per 6-week course. The 16-h were allocated to four cases each distributed as two 2-h sessions in four of the six course weeks. There was no change to content volume as material previously delivered in lecture but not covered in the new sessions was interspersed across the problem-based learning curricular component or independent learning [For a full description of VTCSOM curriculum (LeClair and Binks, 2019)]. In the classroom, learners were expected to integrate material within and across disciplines with expert guidance to develop a deeper approach to learning and practice construction of cognitive schema. The expertise of the educators in the classroom was aligned with the content being covered and each case typically involved 3 to 5 faculty. All the faculty present were involved with developing the cases prior to class with a synergistic mindset to promote integration–we believe this factor was utterly crucial.

### 3.1 Content selection and case-development

**FIGURE 2:**
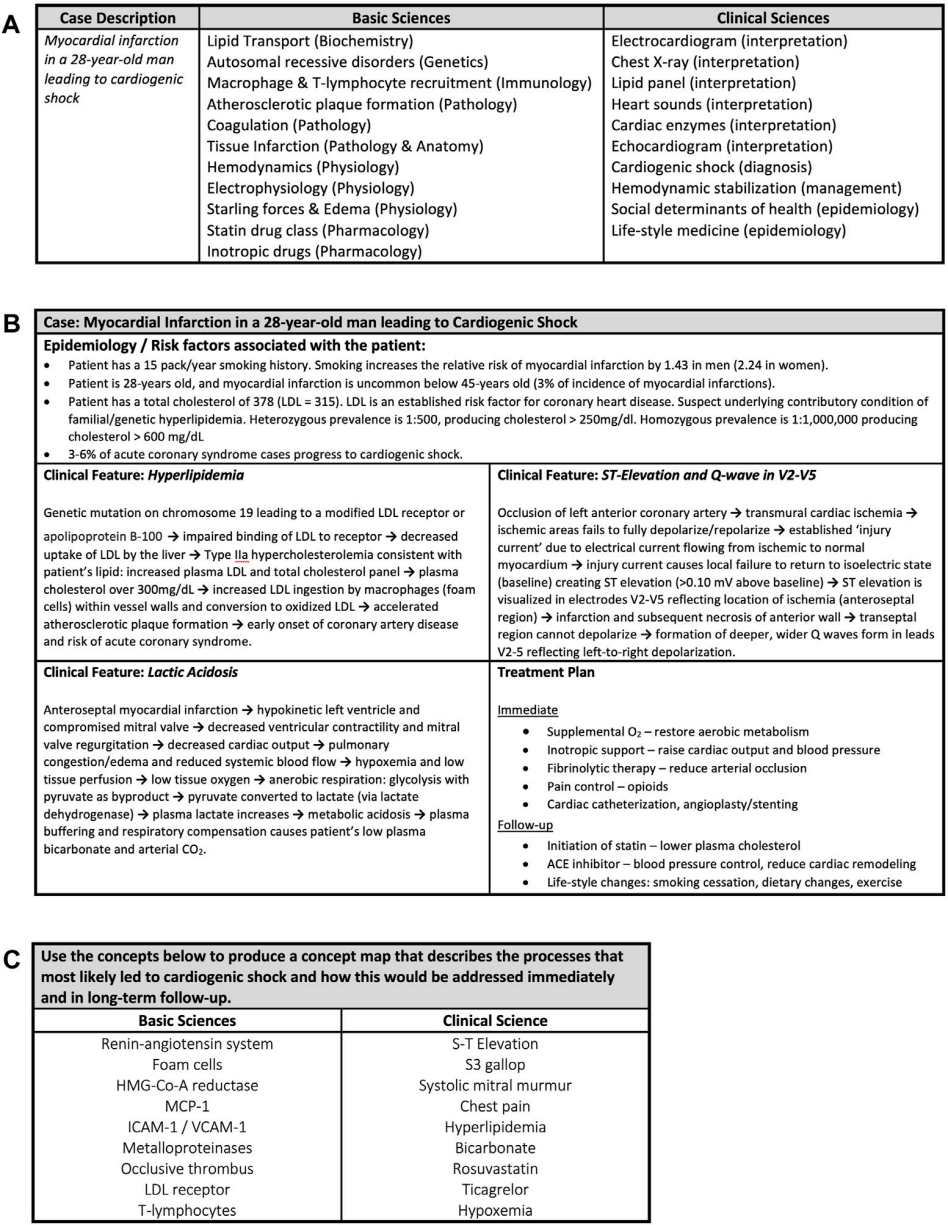
Examples of class planning and delivery material. **(A)** An example of a developed clinical case and the basic science and clinical content that were integrated. For each basic and clinical science concept the associated discipline or skill is shown in parentheses. **(B)** An example of a completed illness script for a clinical case of myocardial infarction. Learners completed the illness script after determining the correct final diagnosis with educator facilitation. The elements for completion are the underlying epidemiological and risk factors associated with the patient in the case, three clinical features that appeared in the case and a treatment plan. In this particular case the learners were asked for an immediate treatment plan to stabilize the patient and a follow-up plan for out-patient care. If the case required further enquiry, then “further diagnostics” could be included in the treatment plan. The → can be read as “leads to”. LDL = Low Density Lipoprotein. **(C)** An example of a concept map prompt for a case of myocardial infarction (MI) that leads to cardiogenic shock. The prompt asks learners develop a concept map that integrates the underlying basic science processes that led to the patient’s MI and shock and connect those mechanisms to the clinical signs, symptoms and data included in the case. Integration of the treatment plan provides opportunity for the learner to demonstrate the reasons and rationale for their choices for case management. The concepts were identified by degree of intrinsic cognitive load.

Cases were selected and developed using several criteria. Firstly, the disease-state associated with the case needed to be significant (LeClair et al., 2018) in terms of clinical prevalence or occurrence on board exams. Secondly, the case needed to involve complex and demanding concepts [i.e., with high intrinsic cognitive load (Paas et al., 2003)] so that difficult concepts were addressed in the classroom with peer and faculty facilitation, while less demanding concepts could be mastered independently. Lastly, we chose cases that provided ample opportunity to integrate across disciplines (Goldman and Schroth, 2012). While this third criterium was usually the easiest to meet, we did not restrict our cases to one time point (allowing us to integrate aspects of disease progression, e.g., chronic inflammation progressing to fibrosis) or a single disease (few patients present with only one condition). An example of a case that met these criteria (myocardial infarction in a 28-year-old man) and the disciplines that were integrated within it are shown in Figure 2A. To maintain students’ trust and engagement, the cases had to be complex and clinically plausible; for which, the role of the physicians was crucial. When recruiting faculty to engage in case writing and the classroom, each was approached with a rationale for the curricular change, an explanation of the methods, and an outlined lesson plan. By working with educators who embraced the goal of trans-disciplinary integration, the development of cases was synergistic, respectful and a learning opportunity for all involved.

### 3.2 Independent learning and problem based learning

To avoid biasing the students’ approach to the case, independent learning objectives were distributed on a week-by-week basis, rather than the session-by-session basis that has previously been used in the prior lecture-based curriculum. Educators identified or developed learning resources [e.g., (Binks, 2022a; Binks, 2022b)] that addressed the learning objectives and they were also distributed on a week-by-week basis. Resources were selected or designed to promote engagement and encourage the students to come to the class prepared with a basic understanding of the week’s content [reviewed in (Binks and LeClair, 2018)]. VTCSOM has a large problem based learning component of the delivery (separate from what is presented here) and other topics were woven into the cases presented during that time.

### 3.3 Case day 1: Differential diagnosis & illness scripts (2-h)

Listening to a real Illness Script being activated: During the first hour, the learner groups were progressively presented with the clinical case. The presentation was structured to mimic a realistic clinical encounter as much as was feasible; first the patient’s appearance and chief complaint were given, then vital signs and medical history, then patient interview, diagnostic test results, and so on. At each step of the case presentation, the small groups generated, refined and justified a differential diagnosis. After each group had reported back to the class, a physician gave their interpretation of the same data and explained how they integrated it to generate their own differential diagnosis. In short, we asked the expert to verbalize their illness script. To help the learners reflect on their own processing, the physician also provided feedback on the groups’ interpretations and differential diagnoses.

Producing an integrated representation of an Illness Script: Groups spent the next hour completing a written illness script (Lubarsky et al., 2015; Moghadami et al., 2021). The illness script prompt (a five-box table, Figure 2B) asked each group to determine, 1) the underlying risk factors and epidemiology for the patient’s condition, 2) the pathological mechanisms underlying three signs or symptoms that arose in the case presentation and 3) their treatment plan and/or further diagnostic tests. The illness script prompt served as an educator-provided framework that the learners completed by integrating their collective knowledge. Explaining the underlying mechanisms of signs and symptoms involved integration of basic science disciplines with clinical science, but the signs and symptoms that educators chose determined the degree of knowledge integration needed to give a full explanation. For example, in the case of myocardial infarction leading to cardiogenic shock shown (Figure 2B), explaining the patient’s high blood lactate as one of the clinical signs required integration of clinical skills, physiology and biochemistry.

### 3.4 Case day 2: Secondary levels of integration with concept mapping (2-h)

With the illness script completed and reviewed by content experts, the second session focused on adding concepts to the learners’ developing schema. Concept mapping (Novak, 2010) proved an ideal tool to integrate between elements of the illness script and additional concepts embedded in the same case. As a visual representation of concepts and their interconnections, construction of a concept map allowed the learners to explore and share their knowledge in a constructionist learning approach (Laight, 2004; Kostovich et al., 2007; Daley and Torre, 2010; Richards et al., 2013; Vink et al., 2016), establish deeper levels of understanding and develop critical thinking (González et al., 2008; Taylor and Littleton-Kearney, 2011; Yelich Biniecki and Conceição, 2016).

Fuller explanations of concept mapping methods are available (LeClair and Binks, 2020), but in brief, educators provide a framework of core concepts for groups to include on their concept maps within a “prompt” of approximately ten basic science and ten clinical science concepts (see example in Figure 2C). Like the selection of signs and symptoms for the illness scripts, the concept map “prompt” provided learners with content boundaries and stemmed inclusion of tangential topics (LeClair and Binks, 2020). Educator-selected concepts could not be connected directly without the learners adding their own intermediate concepts to make meaningful interconnections and achieve a high level of integration. These interconnecting concepts could arise from the individual’s own exploration, or result from social learning with contributions from peers, but adding them to an existing cognitive schema promotes meaningful learning (Yelich Biniecki and Conceição, 2016).

Groups completed their map electronically using software (CMap Tools) specifically designed for shared construction and visualization of concept maps (Cañas et al., 2004). Being able to see each group’s large computer monitor, educators could observe progress and identify significant content deviations or egregious errors. This allowed facilitation (rather than content teaching) on an *ad hoc* basis and just-in-time development of a focused summary of the case in the final 15-min (LeClair et al., 2018), i.e., common errors or questions that arose frequently during the session were addressed by the content expert within the summary. Both products, illness script and concept map were graded using a specifications grading model addressing both content and process [e.g., group dynamics (Hopper et al., 2020)]; groups were assigned a single grade for the week.

## 4 Summary

The call to transition to “integrated curricula” in medical education (Muller, 1984; Anderson and Swanson, 1993; U.K. General Medical Council, 1993) and provide efficiency and context-based delivery is based on the educational benefits, including promoting deeper and holistic learning (Harden et al., 1984) and improving diagnostic competence (Schmidt et al., 1996). In our program, we took this as an opportunity to implement a trans-disciplinary curricular model in the second year of our pre-clerkship curriculum to bring added value to the classroom beyond content delivery.

Our goal was to integrate discipline based content to assist learners in the transition from novice-to expert-thinking by developing cognitive scripts. We chose pedagogical methods (illness scripts and concepts maps) that reflected the intended changes to learning behavior and the long-term goal. The concept mapping sessions in particular promoted more curious “why” questions and fewer terminal “what” questions (Schmidmaier et al., 2013) within the groups. Determining the efficacy of our content integration model will require longitudinal measurement and tracking clerkship and downstream board exam performance will provide insights into any long-term impacts. The clinical case-based approach made content selection and discipline integration relatively straight forward when physiology and pathophysiology were considered as manifestations of other biochemical, immunological, pharmacological (…etc.) processes or disruptions. Beyond these elements however, the consideration and alignment of non-content elements into an integrated curricular model was equally important. While the pedagogies and assessment tools we adopted could be transferred to any program setting, we suggest it is critical to consider these design elements and programmatic boundaries that are specific to the environment and learner.

## Data Availability

The original contributions presented in the study are included in the article/Supplementary Material, further inquiries can be directed to the corresponding author.
